# The Dental Profession in Europe

**Published:** 1855-04

**Authors:** 


### THE DENTAL PROFFSSION IN EUROPE.

The following letter from Dr. E. Parmly, will be read with interest by many of the Profession. We are truly glad that one who has labored so long and efficiently in New York, is now able to take a little relaxation, and as we hope re-invigoration by an European tour. The fact which Dr. Parmly states, that the Profession in Europe do not wear themselves out so fast as we do in this country, is worthy of some thought. We are either in "haste to be rich," or we are not remunerated enough to take our ease. Either is a dilemma which we should avoid. Do well what we do and take time for rest! should be our motto, and yet it is often hard to follow. We hope that Dr. Parmly will be much invigorated by his trip, and long contribute as he has heretofore done to the elevation of Dental Surgery in the United States.

No. 16 Place Vendome, Paris.

Dr. A. Hill,—My Dear Sir,—I have not been unmindful of the request you made of me to inform you of matters of interest relating to our Profession, nor of my promise to do so; but the rapid traveling from one city, and one country to another, our short stays at each place, our daily visiting new beauties and wonders of nature and art on a large scale, has prevented my making any particular inquiry into the minute objects of our Profession.

During my short stay in London I had most agreeable interviews with some of the most highly distinguished gentlemen of our Profession there. Mr. Cartwright, who has been the able representative of practical Dentistry in England for some forty years, is still in practice, and although he had gained a high reputation and a large practice when I practised in London, thirty-four years ago, he is now actively engaged, and has a youthful appearance compared with one of "whitened locks" much younger than himself who has been engaged in professional labor a greater part of the time since that period, in New York. Mr. Thomas Bell, who has also been a "hard worker," and who has done more for the Profession in England in years past, by his valuable writings and lectures, than any other man in England, although my senior by several years, has worn better, and is a much younger looking man. I mention these two instances in this way, to prove that Professional men in England do not wear by professional labor as rapidly as we do in America.

There are some in the Profession, and many out of it, among his old patients, that will be glad to hear that Mr. Charles Newton, who was in New York when I went there, and was as an operator taking both the mechanical and surgical departments of our art, the cleverest man I had ever known, or ever have known even up to the present day, is still living and in good health. I could not ascertain until the morning I left England where he could be found, therefore did not see him, though to have done so would have given me very great pleasure.

Messrs. Rogers, Tomes, and Biggs, all of whom are highly distinguished men in London, I also had a most agreeable interview with, at the house of Mr. Tomes, who is well known among us for his valuable writings. The manner in which Mr. T. talked of his various modes of practice and exhibited to me his improved instruments (and he has the best assortment of forceps I have ever seen) makes the narrow-minded exclusiveness of some of our own men who deal in mysteries and secrets appear smaller than ever by way of contrast.

There are several American Dentists in London, two of whom I had the pleasure of seeing, whose personal courtesy I not only feel happy in acknowledging, but feel much happier in saying that the professional merits of Mr. Ballard, and also the merits of Mr. Rapn (the latter of whom I met at Mr. Tomes') are owned and appreciated there by the Profession. It is a pleasant thing to have either social or professional intercourse with such men as give character and standing to our calling in England, and I regret that my stay was so limited in London that I could not avail myself of the social courtesies so cordially extended.

As you are the editor of one of the American journals, I will take occasion to repeat to you a remark made by one of the above named English gentlemen. He said, "I shall give up all American journals, as they admit so much into them that is low, ungentlemanly, and abusive of each other, that I have a feeling of dread and disgust almost every time I open one." I felt too keenly the truth and justice of the remark to contradict it, not knowing exactly how far he included myself in the language I have used in exposing deception and imposition, and entirely condemning the whole system of Amalgam practice as followed by many, while at the same time I have endeavored to establish a uniform system of practice without Amalgam, which as a whole I believe all agree with me in sustaining, and as my opponents have never said aught against the practice I recommend, I conclude they have nothing to say. This same gentleman, although, he contends for the right to use Amalgam in a very limited number of cases, I was glad to learn from him that he condemns as strongly as I do the wholesale and distributive use that is made of it in both England and America, and I cannot yet perceive how any true lover of Dental science can do otherwise.

There are scattered over the Continent several American Dentists. In Paris there are now four in practice. The

Messrs. Evans, Dr. Hoener from Philadelphia, and a Mr. Potter from New Orleans, all I believe doing well. Mr. T. W. Evans being the Dentist to several of the European Courts, is frequently sent for by them, and is now on one of his Professional visits to Germany.

Mr. Brewster, who is the pioneer and really the establisher of American Dentistry on the continent of Europe, is now in Paris, but not in practice, as he is precluded by certain stipulations in his negotiations with Mr. Evans, who succeeds him perhaps in the most lucrative practice ever established on the Continent by any one person.

